# Are changes in body-mass-index associated with changes in depressive symptoms? Findings of a population-based longitudinal study among older Germans

**DOI:** 10.1186/s12888-018-1748-1

**Published:** 2018-06-08

**Authors:** André Hajek, Hans-Helmut König

**Affiliations:** Department of Health Economics and Health Services Research, Hamburg Center for Health Economics, University Medical Center Hamburg-Eppendorf, Hamburg, Germany

**Keywords:** Depressive symptoms, BMI, CES-D, Curvilinear effect, Longitudinal study

## Abstract

**Background:**

The longitudinal relationship between BMI and depressive symptoms is not well understood. Therefore, we aimed at investigating the long-term association between body-mass-index (BMI) and depressive symptoms among older Germans.

**Methods:**

Data were derived from a population-based longitudinal study of adults aged 40 and above in Germany (German Ageing Survey, DEAS). Four waves (2002–2014) were used. Depressive symptoms was assessed by the Center for Epidemiologic Studies Depression Scale (CES-D). Linear, quadratic and cubic terms were included for self-reported BMI. Fixed effects regressions were used to estimate the predictors of depressive symptoms.

**Results:**

FE regressions showed a curvilinear effect of BMI on depressive symptoms in the total sample and in women, but not men, with significant gender differences. In sum, the greater the extreme of BMI (either higher or lower), the greater the risk for depressive symptoms in the total sample and in women.

**Conclusion:**

Our findings indicate that the effect of BMI on depressive symptoms is by no means simple. The current study highlight the importance of comprehensive treatment of depression, which include management of (extreme) weight to manage depressive symptoms.

## Background

It is expected that the number of individuals with excess weight increases markedly in the next decades as the mean age is expected to increase and the mean body weight gradually rises with age [1]. While there is large evidence that an increased body-mass-index (BMI) is associated with numerous adverse health-related *physical outcomes* such as a decreased physical function [2, 3], cardiovascular diseases, osteoarthritis, or cancer [4, 5], little is known about the consequences of weight changes on *psychological outcomes* such as depressive symptoms.

Cross-sectionally, the association between BMI and depressive symptoms has widely been studied, with mixed evidence. While some studies found a negative association between excess weight and depressive symptoms in older adults [6, 7], others found no association [8], whereas others found a positive association [9]. Equivocal results might be mainly explained by differences in statistical methods, culture and attitudes towards excess weight. The mixed evidence might also be explained by the curvilinearity in the relationship between BMI and depressive symptoms.

However, as already argued by Roberts and colleagues [10], longitudinal studies are needed to get insights into the causal relationship between BMI and depressive symptoms. By using longitudinal data, intraindividual changes can be investigated. Moreover, the problem of omitted variable bias can be solved [11]. Thus far, only a few longitudinal studies exist examining the effect of BMI on depressive symptoms among older adults [12–15], mainly indicating that excess weight increased the risk of subsequent depressive symptoms.

In addition, the *longitudinal relationship* between BMI and these outcome variables is not well understood. For example, do we become more depressed as we lose or increase weight? Or are changes in depressive symptoms not associated with weight changes? Or are changes in weight related to changes in depressive symptoms in a curvilinear pattern, with individuals becoming underweight and overweight scoring worse? Based on previous studies showing that underweight as well as overweight are associated with increased depressive symptoms [16, 17], we hypothesize that the greater the change to the extreme of BMI, the greater the risk of an increase in depressive symptoms. More specifically, factors associated with obesity such as poor body image, maladaptive eating behaviors or avoidance of physical activity might explain the relationship between obesity and depressive symptoms [18]. The relation between low weight and depressive symptoms might be explained by lifestyle factors (e.g., alcohol consumption) and chronic illnesses [19]. In addition, biological factors such as high density lipoprotein (HDL) cholesterol might play a role in this relationship [19].

Furthermore, we hypothesize that the relationship between changes in BMI and changes in depressive symptoms is stronger in women [20–22] than in men since extreme BMI values might carry greater stigma in women [23].

Consequently, we aimed at investigating the longitudinal relationship between intraindividual changes in BMI and intraindividual changes in depressive symptoms by using a large population-based longitudinal study of adults aged 40 and over (2002–2011) in Germany. Fixed effects regressions were used (with linear [BMI], quadratic [BMI*BMI] and cubic terms [BMI*BMI*BMI] for BMI) to examine whether changes in BMI are associated with changes in depressive symptoms, adjusting for several covariates. Furthermore, by using FE regressions, time-constant factors (both, unobserved and observed) were taken into consideration.

## Methods

### Sample

Currently, the German Ageing Survey (DEAS) has completed five survey waves, beginning in 1996. It is a nationally representative sample of the population aged 40 and over living in private households. The sample for the current study was drawn by a national probability sampling stratified by age, sex, and place of residence. Participants were interviewed by trained staff using a standardized questionnaire.

As depressive symptoms were quantified from the second wave onwards, we used data from wave 2 (2002) to wave 5 (2014). In the second wave 5194 subjects were interviewed, whereas 8200 subjects were interviewed in the third wave (2008). 4855 individuals were included in the fourth wave (2011), and 10,325 individuals participated in the fifth wave. The large discrepancies between the sample sizes by survey year can be explained by the introduction of new samples. For example, 6205 subjects were interviewed for the first time in the third wave and 1995 subjects have been interviewed in former waves (994 in the first wave and 1001 in the second wave). In the fourth wave, only panel-willing participants (individual willing to participate in a future wave) of the previous waves (wave 1: 1040; wave 2: 957; wave 3: 2858) have been re-questioned. Thus, the fourth wave is a pure panel survey. While 6002 individuals were interviewed for the first time in the fifth wave, 4323 individuals have been interviewed in former waves (888 in the first wave, 866 in the second wave and 2569 in the third wave). The baseline samples provide information to describe and investigate the current situation and social changes over time. In 1996, individuals of the birth cohorts 1911–1956 were used (2002: 1917–1962; 2008: 1923–1968; 2014: 1929–1974). A very recent study by Klaus et al. [24] displayed the sample design of the German Ageing Survey in detail. Moreover, detailed figures were given for the DEAS baseline samples 1996, 2002, 2008 and 2014 and the longitudinal samples 2002, 2008, 2011 and 2014.

Data were gathered from the German population living in private households aged 40 and over in 1996, whereas from 2002 even non-German nationals were included (random sampling of non-Germans nationals in the birth cohorts 1917–1962). The main reasons for a lack of follow-up data were refused further participation and health reasons. More details with regard to the sample composition and the sampling frame were provided elsewhere [25]. Prior to the interview, written informed consent was given. Individuals received a small incentive for participation (1996, 2002: telephone card; 2008: stamp album; 2011: €10) to increase response rate [26]. This was also done to increase response rate in groups, which are often underrepresented in surveys (e.g., low educated individuals or singles) [27].

### Dependent variable

Depressive symptoms were assessed by means of the short version [28] of the Center for Epidemiologic Studies Depression Scale (CES-D, 15 items) [29], a well-validated scale with good psychometric properties [30]. The scale is the sum of all 15 items, ranging from 0 to 45 (high values indicate more depressive symptoms).

### Independent variables

Our main variable of interest, BMI (linear, quadratic and cubic terms) was computed as self-reported weight in kilograms divided by height in square meters (kg/m^2^). Quadratic and cubic terms were used to explore the possible nonlinear relationship between BMI and depressive symptoms over time. Furthermore, control variables were integrated in the main model: age, marital status (Ref.: married, living together with spouse; married, living separated from spouse; divorced; widowed; never married), (log) monthly household net income in Euro, employment status (Ref.: working; retired; other: not employed), and comorbidity (number of illnesses, e. g. osteoporosis or cancer). Moreover, the validated Rosenberg scale [31], consisting of ten items (from 1 = “strongly agree” to 4 = “strongly disagree”), was used to assess self-esteem. It was included as a potential confounder in our main regression model because it might affect the outcome variable. An index score was developed by averaging the items (higher values indicate higher level of self-esteem). Cronbach’s alpha was .84. In addition, the number of important people in regular contact (0–9) was included.

Additionally, the time-invariant variable education was reported to describe the sample. It was measured by using the International Standard Classification of Education (ISCED-97) [32], with low (0–2), medium (3–4) and high education (5–6). We performed robustness checks by running regression models (sensitivity analysis) where we additionally controlled for functional health because it might affect the outcome variable. This was measured by the subscale “physical functioning” of the 36-Item Short Form Health Survey (SF-36) [33]. The individuals rated impairments in ten activities of daily living (e.g., bathing or lifting) on a three-point scale [34]. The items were transformed into a single scale (from 0 = worst score to 100 = best score). In addition, further analysis was conducted with BMI categories using the WHO thresholds: underweight (BMI < 18.5 kg/m^2^), normal weight (18.5 kg/m^2^ ≤ BMI < 25 kg/m^2^), overweight (25 kg/m^2^ ≤ BMI < 30 kg/m^2^), and obesity (BMI ≥ 30 kg/m^2^). While the WHO thresholds additionally differentiate between obese grade I (30 kg/m^2^ ≤ BMI < 35 kg/m^2^), obese grade II (35 kg/m^2^ ≤ BMI < 40 kg/m^2^), and obese grade III (BMI ≥ 40 kg/m^2^), it is worth stressing that these additional classifications were not used for reasons of simplicity.

Moreover, additional regression models were computed as follows: Depressive symptoms were replaced by log depressive symptoms to account for the skewness (linear FE regressions). In addition, depressive symptoms were considered as count data. Thus, FE Poisson regressions and FE negative binomial regressions were computed.

Furthermore, depressive symptoms were replaced by depression (CES-D ≥ 18 [30]). Consequently, conditional FE logistic regressions were used.

Moreover, self-esteem was excluded from our main regression model in further sensitivity analysis. In addition, very extreme BMI values were excluded from FE regression analysis in another sensitivity analysis.

### Statistical analysis

First, descriptive characteristics and pattern of change for individuals included in linear FE regressions were reported. Second, pairwise correlations were reported to yield insights into the relation between BMI and depressive symptoms. Third, longitudinal regression analysis (linear FE regressions) was performed.

One of the main advantages of longitudinal data is that time-constant unobserved factors can be taken into account. This is crucial because these time-constant unobserved factors such as genetic disposition, or pessimism are often systematically correlated with the predictors [11]. If this correlation is present, not only pooled ordinary least squares (OLS) estimates but also random effects (RE) strategies are inconsistent [35]. Contrarily, fixed effects (FE) regressions provide consistent estimates (under the assumption of strict exogeneity) even if time-constant unobserved factors are correlated with the predictors [35]. Actually, some recent studies [36–38] have shown that FE regressions are the method of choice with depressive symptoms as outcome variable. The strict exogeneity assumptions means that FE estimates are biased under endogeneity. Endogeneity in turn can have different sources: (1) unobserved time-varying confounders, (2) reverse causality, simultaneity (Y also influences X), (3) measurement errors and (4) endogenous selection bias. Please see Brüderl and Ludwig for further details [39].

One of the main goals was to provide consistent estimate under rather weak assumptions [39]. Consequently, FE regressions were used. Sargan-Hansen tests supported this choice. A Sargan-Hansen test is a Hausman test with robust standard errors. The intuition of this test is that FE estimates are consistent. If the estimates of the RE regressions do not differ systematically, then RE regressions can be used (because they are more efficient since they use within- and between-information). The Sargan-Hansen test statistic was 214.1, *p* < .0001 (main model, total sample), suggesting that there were systematic differences between the coefficients observed using the FE and the RE regressions. In addition, the strengths and limitations of the FE model are discussed by Brüderl and Ludwig [39].

FE regressions solely use intraindividual changes (changes within individuals over time). Hence, the FE-estimator is also called ‘Within-estimator’. Thus, the association between intraindividual changes in BMI and intraindividual changes in depressive symptoms was examined. However, for linguistic reasons, seemingly causal terminology was used in the current study, though this should be interpreted with great caution [39–41]. Please see the strengths and limitations section for further details. Further technical details are described elsewhere [35, 42, 43].

According to Cameron and Trivedi [35], it was tested whether the depressive symptoms variable has enough within variation (‘xttab’ and ‘xttrans’ in Stata) to obtain precise estimates. For example, in each wave only about 26% of the individuals with no depressive symptoms (= 0) in the data had no depressive symptoms in the next year. The remaining 74% became 1 or more. Thus, there is enough within-variation over time.

It is worth noting that individuals where BMI does not change did not contribute to the FE-estimator (no control group). However, as stated by Brüderl and Ludwig [39], by including age in our FE regression model (a so-called two-way FE regression model), the control group information is used for estimating the age effects. Thus, a general recommendation is to include age or period effects in FE regression analysis [39].

## Results

### Sample characteristics and pattern of change

Because we were interested in changes within individuals over time, only individuals were included in the FE estimates if they had changes in the outcome variable between the second and the fifth wave. Descriptive statistics for individuals included in FE regressions (total sample) are shown in Table 1. Data of 12,793 individuals were used. Besides, it is worth stating that the Stata command for FE regressions include individuals with one observation in computing the number of observations because these individuals deliver information about the between R^2^, the overall R^2^, the variance components, the constant, as well as the correlation between individual effects constant over time and the independent variables. However, it is also worth emphasizing that this does not affect the beta-coefficients and the standard errors. Thus, it is worth stressing that individuals with only one observation did not contribute to the beta-coefficients and the standard errors of the FE regression analysis. Please see for further details [44]. We used the ‘xtreg’ command with the ‘fe’ option for computing linear FE regressions.

**Table 1:** Sample Characteristics for individuals included in fixed effects regressions (2002–2014, pooled)

Male: N (%)	10,142 (51.2)
Education: Low education (ISCED-97: 0–2): N (%)	1305 (9.4)
Education: Medium education (ISCED-97: 3–4): N (%)	7320 (52.9)
Education: High education (ISCED-97: 5–6): N (%)	5219 (37.7)
Age: Mean (SD), Range	63.5 (11.4), 40–95
Married, living together with spouse: N (%)	14,187 (71.6)
Employment status: Working: N (%)	7077 (35.7)
Employment status: Retired: N (%)	10,543 (53.2)
Employment status: Other: N (%)	2206 (11.1)
Number of important people in regular contact: Mean (SD), Range	4.9 (2.7), 0–9
Self-esteem (Rosenberg scale)	3.4 (0.4), 1.2–4
Number of chronic diseases: Mean (SD), Range	2.5 (1.9), 0–11
BMI: Mean (SD), Range	26.7 (4.4), 15.2–69.1
Depressive symptoms (CES-D): Mean (SD), Range	6.7 (6.1), 0–45
Observations	19,826
Number of individuals	12,793

Concerning the time-constant variables, which were not included in FE regressions as predictors, the majority was male (51.2%) and had a medium educational level (52.9%). The mean age was 63.5 years (±11.4 years, 40–95 years). Most of the individuals were married, living together with spouse (71.6%) and retired (53.2%). The mean number of important people in regular contact was 4.9 (±2.7), the mean self-esteem score was 3.4 (±0.4), the mean number of chronic diseases was 2.5 (±1.9), the mean BMI was 26.7 (±4.4, 15.2–69.1; men: 27.1 ± 4.0, 15.2–69.1; women: 26.3 ± 4.8, 15.2–68.8) and the mean depressive symptoms score was 6.7 (±6.1; skewness: 1.7).

The mean level of changes from wave 2 to wave 5 in BMI and depressive symptoms were positive in depressive symptoms (indicating more depressive symptoms over time) and BMI, i.e. overall mean level of change in depressive symptoms was 0.08 (±6.04) and overall mean level of change in BMI was 0.45 (±3.72).

### Correlations

We computed cross-sectional Pearson correlations (Table 2) in order to gain some insight into the relationships between the variables.

**Table 2:** Pairwise cross-sectional correlations (with Bonferroni correction for multiple comparisons; 2002–2014, pooled)

	Depressive symptoms (CES-D)	Age	Marital status (Ref.: married, living together with spouse)	Employment status: retired (Ref.: Working)	Employment status: other	Number of important people in regular contact	Self-esteem (Rosenberg scale)	Number of chronic diseases	BMI
Depressive symptoms (CES-D)	1								
Age	0.0145	1							
Marital status (Ref.: married, living together with spouse)	0.128***	0.0683***	1						
Employment status: retired (Ref.: Working)	0.0276**	0.772***	0.0442***	1					
Employment status: other	0.0602***	−0.155***	0.00624	−0.377***	1				
Number of important people in regular contact	− 0.0739***	− 0.107***	− 0.114***	− 0.0784***	− 0.0163	1			
Self-esteem (Rosenberg scale)	−0.395***	−0.0343***	− 0.0699***	−0.0390***	− 0.0640***	0.0555***	1		
Number of chronic diseases	0.280***	0.365***	0.0607***	0.306***	−0.0401***	−0.0140	− 0.236***	1	
BMI	0.0438***	0.0412***	−0.0214+	0.0591***	0.00881	−0.0141	−0.0442***	0.185***	1
Observations	19,826								

*** *p* < 0.001, ** *p* < 0.01, * *p* < 0.05, + *p* < 0.10 (listwise deletion was used)

Depressive symptoms were significantly associated with marital status (*r* = .13, *p* < .001), employment status (retired: *r* = .03, *p* < .01; other: .06, p < .001), the number of important people in regular contact (*r* = −.08, *p* < .001), self-esteem (*r* = −.40, *p* < .001), the number of chronic diseases (*r* = .28, *p* < .001) and BMI (*r* = .04, *p* < .001), whereas depressive symptoms were not significantly associated with age.

### Regression analysis

**Fig. 1:**
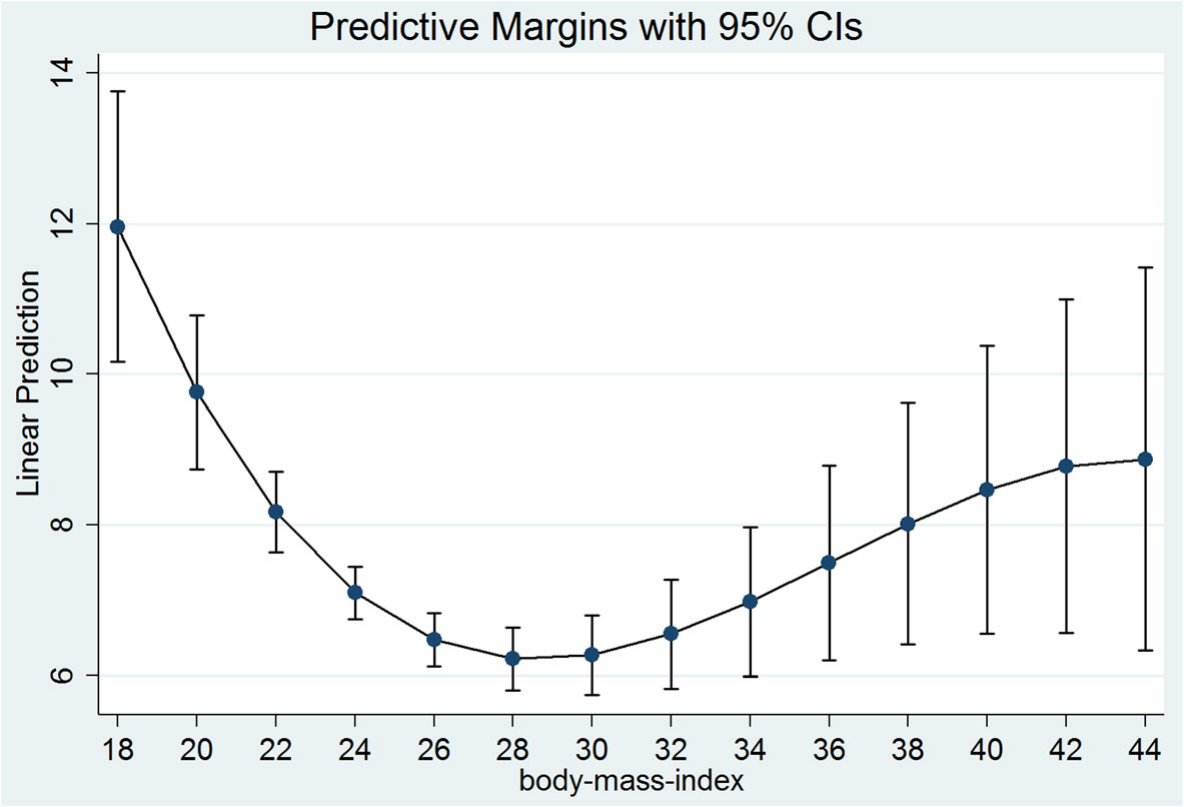
Relation between depressive symptoms (CES-D) and BMI among women

**Fig. 2:**
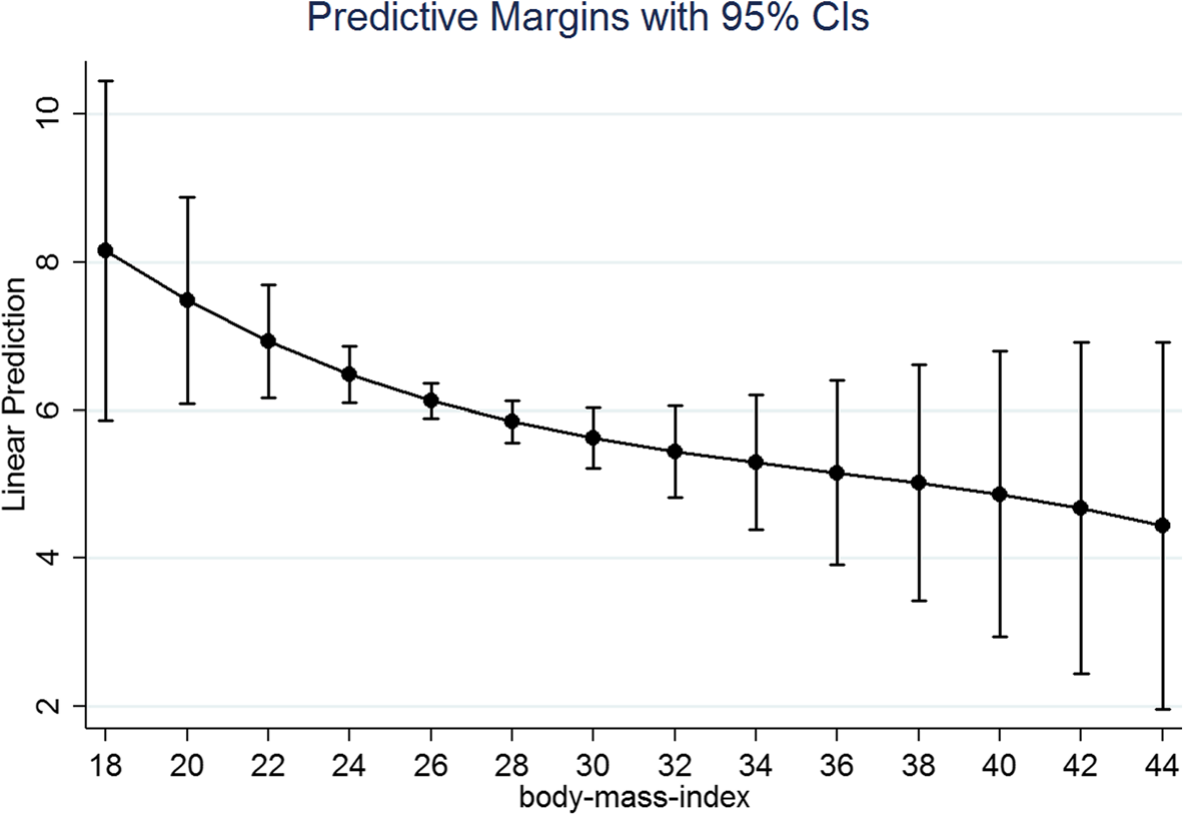
Relation between depressive symptoms (CES-D) and BMI among men

In Table 3, we used three model specifications for the total sample (columns 1–3), for men (columns 4–6) and for women (columns 7–9). In the first model specification, we included only the linear BMI term (besides potential confounders; column 1, 4 and 7). In the second model specification, we additionally used a quadratic term for BMI (column 2, 5 and 8). In the third model specification, a cubic term for BMI was included (column 3, 6 and 9). FE regressions revealed that the effect of BMI on depressive symptoms is curvilinear in the total sample and in women, but not men. To ease the interpretation, our (sex-specific) analysis is illustrated using “marginsplot” in Stata (Figs. 1 and 2). According to Fig. 1, there seems to be a curvilinear effect in women, i.e. the curve illustrates a steep decline in the outcome measure and reaches a plateau (normal weight and overweight). After that (excess weight), depressive symptoms increases. In contrast to that, there seems to be an almost linear relationship between depressive symptoms and BMI in men. More specifically, the greater the BMI, the lower depressive symptoms. There was a significant interaction between BMI with gender (linear: *p* < .05; quadratic: *p* < .05; cubic: *p* < .05).

**Table 3:** Predictors of depressive symptoms (CES-D) – results of fixed effects regressions (total sample; men; women)

	(1)	(2)	(3)	(4)	(5)	(6)	(7)	(8)	(9)
Independent variables	Depressive symptoms	Depressive symptoms	Depressive symptoms	Depressive symptoms - Men	Depressive symptoms - Men	Depressive symptoms - Men	Depressive symptoms - Women	Depressive symptoms - Women	Depressive symptoms - Women
Age	−0.0185	−0.0172	−0.0201	0.00932	0.00943	0.00813	− 0.0504*	−0.0473*	− 0.0501*
	(0.0149)	(0.0150)	(0.0150)	(0.0187)	(0.0187)	(0.0187)	(0.0235)	(0.0235)	(0.0236)
Other marital statuses (Ref.: Married, living together with spouse)	0.480	0.461	0.461	0.133	0.129	0.131	0.904	0.867	0.849
	(0.441)	(0.441)	(0.440)	(0.511)	(0.511)	(0.511)	(0.693)	(0.690)	(0.689)
Retired (Ref.: Working)	−0.364	−0.372	−0.386	−0.306	−0.309	− 0.315	− 0.459	−0.453	− 0.477
	(0.245)	(0.244)	(0.243)	(0.286)	(0.286)	(0.287)	(0.402)	(0.399)	(0.396)
Other: not employed	0.260	0.265	0.263	0.261	0.264	0.271	0.197	0.199	0.173
	(0.285)	(0.284)	(0.283)	(0.372)	(0.372)	(0.371)	(0.422)	(0.420)	(0.418)
Household net equivalent income	−4.92e-06	−1.26e-06	4.66e-06	−3.11e-05	−3.07e-05	−2.92e-05	1.55e-05	2.33e-05	3.37e-05
	(4.47e-05)	(4.44e-05)	(4.39e-05)	(6.36e-05)	(6.37e-05)	(6.38e-05)	(7.20e-05)	(7.17e-05)	(7.05e-05)
Number of important persons in regular contact	−0.0142	−0.0132	−0.0124	−0.00162	−0.00173	−0.000587	−0.0289	− 0.0241	−0.0285
	(0.0228)	(0.0228)	(0.0227)	(0.0279)	(0.0279)	(0.0279)	(0.0365)	(0.0365)	(0.0363)
Self-esteem (Rosenberg scale)	−3.204***	−3.191***	−3.171***	−2.894***	−2.893***	−2.888***	− 3.465***	−3.429***	− 3.399***
	(0.273)	(0.272)	(0.272)	(0.317)	(0.317)	(0.317)	(0.428)	(0.426)	(0.424)
Total number of physical diseases	0.291***	0.288***	0.281***	0.338***	0.337***	0.334***	0.231**	0.231**	0.224**
	(0.0552)	(0.0551)	(0.0549)	(0.0707)	(0.0705)	(0.0702)	(0.0851)	(0.0847)	(0.0843)
BMI	−0.119**	−0.795***	−3.791***	−0.136*	−0.262	−1.291	−0.0963+	−1.204***	−5.766***
	(0.0395)	(0.237)	(0.917)	(0.0561)	(0.257)	(1.288)	(0.0556)	(0.327)	(1.338)
BMI^2^		0.0111**	0.107***		0.00206	0.0342		0.0183***	0.167***
		(0.00396)	(0.0275)		(0.00397)	(0.0377)		(0.00554)	(0.0416)
BMI^3^			−0.000970***			−0.000319			−0.00154***
			(0.000261)			(0.000346)			(0.000415)
Constant	20.82***	30.60***	60.77***	18.18***	20.03***	30.66*	23.31***	39.04***	84.03***
	(1.728)	(3.800)	(9.927)	(2.240)	(4.400)	(14.19)	(2.552)	(5.252)	(14.07)
R^2^	0.045	0.047	0.049	0.050	0.050	0.050	0.045	0.050	0.054
Observations	19,826	19,826	19,826	10,142	10,142	10,142	9684	9684	9684
Number of Individuals	12,793	12,793	12,793	6549	6549	6549	6244	6244	6244

Beta-Coefficients were reported; Cluster-robust standard errors in parentheses; *** *p* < 0.001, ** *p* < 0.01, * *p* < 0.05, + *p* < 0.10; listwise deletion was used. It is worth emphasizing that the Stata command for FE regression analysis include subjects with only one observation in calculating the number of observations since these subjects deliver information about the variance components, the constant, the between R^2^, the overall R^2^ as well as the correlation between individual effects constant over time and the regressors. However, it does not affect the beta-coefficients and the standard errors

In addition, depressive symptoms significantly increased with decreasing self-esteem and increasing comorbidity in the total sample and in both sexes, whereas depressive symptoms were not significantly associated with marital and employment status, income and the number of important persons in regular contact. Furthermore, depressive symptoms were negatively associated with age in women, but not men.

In sensitivity analysis, depressive symptoms was replaced by log depressive symptoms to account for the skewness. In terms of significance, linear FE regressions also showed a curvilinear effect of BMI on depressive symptoms in the total sample and in women, but not men.

Moreover, depressive symptoms was considered as a count variable. Thus, FE Poisson regressions and FE negative binomial regression models were computed. Again, both statistical models revealed a curvilinear effect of BMI on depressive symptoms in the total sample and in women, but not men.

In further sensitivity analysis, we additionally controlled for functional health. Changes in functional health were associated with changes in depressive symptoms (β = −.07, *p* < .001, in the total sample and in both sexes). In addition, the nature of the relationship between BMI and depressive symptoms remained virtually the same.

In further analysis, BMI was categorized according to the WHO guidelines. While changes in BMI categories were not associated with changes in depressive symptoms, changes from ‘normal weight’ to excess weight were positively associated with changes in depressive symptoms in the total sample and in women.

In another model, depressive symptoms were replaced by depression as outcome measure. Consequently, we used conditional fixed effects logistic regressions because we have a binary outcome variable. In this model, FE regressions revealed a curvilinear effect of BMI on depression in the total sample and in women, whereas no association was found in men. With regard to the clinical significance, it is worth noting that in the first model specification where only the linear BMI term was included (beyond the potential confounders), OR for BMI was .931 [95%-CI: .870–997] in the total sample. This means that if an individual increases BMI by one unit, his or her odds of having a depression are multiplied by .93 (ceteris paribus).

In further regression analysis, self-esteem was excluded from our main regression model because it might be on the causal pathway between BMI and depressive symptoms. Compared with our main model findings remained almost the same in terms of significance and effect sizes.

While all self-reported BMI values were used in main analysis, very extreme BMI values (BMI < 15 kg/m^2^ and BMI > 55 kg/m^2^) were excluded from the FE regression analysis in additional analysis. However, our main results remained virtually the same.

## Discussion

### Main findings

We aimed at investigating the nature of the relationship between BMI and depressive symptoms by using a large population-based longitudinal study of adults aged 40 (2002–2011) in Germany. Our FE regressions showed a curvilinear effect of BMI on depressive symptoms in the total sample and in women, but not men, with significant gender differences. Thus, the greater the extreme of BMI (either higher or lower), the greater the risk for depressive symptoms. This effect is likely to be driven by the effect in women.

### Previous research

Recent studies found that excess weight increased the risk of subsequent depressive symptoms [15, 17]. For instance, by using data from the Health and Retirement Study (1994 to 2010, 6514 community-dwelling older adults), Xiang and An [15] found that overweight and obesity predicted the onset of depression up to 16 years by using time-dependent Cox proportional hazards model. Moreover, obesity had stronger associations with depression in women. This is also in line with another recent cross-sectional study [45]. A possible explanation for the gender differences is that women have stronger genetic disposition to excess weight and depressed mood [45]. In addition, social factors such as greater peer pressures or reduced career opportunities might explain why the relationship between excess weight and depressive symptoms is stronger in women [45]. Furthermore, by using multilevel random intercept mixed models, Chang et al. [12] found that the odds of elevated depressive symptoms significantly increased as BMI increased over time. Their data were derived from a short run (four years) longitudinal study (1496 participants) taking place in twelve rural communities in Missouri, Arkansas, and Tennessee.

Moreover, Kim et al. [16] examined the association between self-reported BMI and depressive symptoms (CES-D, 10 items) from 2006 to 2010 in older adults aged 45 years and above in South Korea (representative data from the Korean Longitudinal Study of Aging). Using cross-lagged panel models, they found that there is an association between underweight and the development of depressive symptoms. In sum, by using panel data methods, we provide new evidence that the greater the extreme of BMI (either higher or lower), the greater the risk for depressive symptoms in the total sample and in women. Thus, it is likely that this effect is driven by the one in women. Besides, results regarding the control variables such as self-esteem are in accordance with previous studies [22, 46].

### Strengths and limitations

Our study is the first study that examined the non-linear (any relationship which is not linear, for example, as BMI increases, the resulting depressive symptoms do not increase proportionally) effect of BMI on depressive symptoms in older adults in Germany. By exploiting panel data methods, unobserved as well as observed time-invariant factors (factors that are time-constant such as sex or country of origin) can be taken into account, leading to consistent estimates under the assumption of strict exogeneity. Reducing the problem of unobserved heterogeneity is a huge advantage compared to cross-sectional studies. More specifically, it is almost impossible to control for differences in genetic dispositions between individuals in large surveys. In FE regressions, one does not have to worry about these differences in time-invariant unobserved factors [39]. Furthermore, data were derived from a large population-based sample of community-dwelling older adults (aged 40 and above). Data from 2002 to 2014 were used. Additionally, it is worth emphasizing that we used a validated measure of depressive symptoms (CES-D).

One limitation is that BMI was self-reported. Hence, the BMI might be biased downwards because individuals tend to underestimate weight and overestimate height [47]. However, as long as this downwards bias is constant within an individual over time, it does not bias linear FE estimates because FE estimates rely exclusively on intraindividual changes over time.

Moreover, we could not distinguish between intentional and unintentional weight loss. Furthermore, due to panel attrition (permanent unit-nonresponse) in the German Ageing Survey [48], our estimates might be biased downwards. Moreover, it might be difficult to generalize findings to, e.g., less educated individuals and individuals with low self-rated health for reasons of sample selection bias. Besides, a simultaneity bias (depressive symptoms affect BMI) cannot be ruled out [49–51]. For example, changes in depressive symptoms could affect the BMI due to a loss of appetite. The simultaneity bias is a typical problem in most cases when observational studies are used. Theoretically, this can be handled by using panel instrumental variable approaches [35]. However, truly exogenous and strong instruments are difficult to identify. Moreover, it rests on some untestable assumptions. As stated by Mouw [52], the researcher has to rely on theoretical justifications. Other available models such as cross-lagged structural equation models have also been criticized for various reasons [39, 52]. Furthermore, other factors such as drugs (antidepressant medications) might play a role in the relationship between BMI and depressive symptoms. However, for reasons of data availability, we were unable to integrate these factors into the model. Nevertheless, we already controlled for numerous important covariates.

## Conclusion

Our data indicate that the impact of BMI on depressive symptoms is by no means simple. The current study highlight the importance of comprehensive treatment of depression, which include management of (extreme) weight to manage depressive symptoms. This might also be important since increased depressive symptoms in older age are positively associated with other adverse health outcomes such as functional impairment [53], frailty [54] or admission to nursing home [55].

## Abbreviations

BMI: Body Mass Index
CES-D: Center for Epidemiological Studies Depression Scale
DEAS: German Ageing Survey
FE: Fixed effects
ISCED: International Standard Classification of Education
OLS: Ordinary least squares
OR: Odds Ratio
RE: Random effects
SF-36: Short Form-36
WHO: World Health Organization
